# The spectrum of eating environments encountered in free living adults documented using a passive capture food intake wearable device

**DOI:** 10.3389/fnut.2023.1119542

**Published:** 2023-05-11

**Authors:** Matthew Breit, Jonathan Padia, Tyson Marden, Dan Forjan, Pan Zhaoxing, Wenru Zhou, Tonmoy Ghosh, Graham Thomas, Megan A. McCrory, Edward Sazonov, Janine Higgins

**Affiliations:** ^1^Department of Medicine, Division of Endocrinology, Metabolism, and Diabetes, University of Colorado Anschutz Medical Campus, Aurora, CO, United States; ^2^Colorado Clinical and Translational Sciences Institute, University of Colorado Anschutz Medical Campus, Aurora, CO, United States; ^3^Research Institute Biostatistics Core, Department of Pediatrics, University of Colorado Anschutz Medical Campus, Aurora, CO, United States; ^4^Department of Electrical and Computer Engineering, The University of Alabama, Tuscaloosa, AL, United States; ^5^Department of Psychiatry and Human Behavior, Weight Control and Diabetes Research Center, Alpert Medical School of Brown University and The Miriam Hospital, Providence, RI, United States; ^6^Department of Health Sciences, Boston University, Boston, MA, United States

**Keywords:** wearable device, eating environment, dietary intake, obesity, passive capture

## Abstract

**Introduction:**

The aim of this feasibility and proof-of-concept study was to examine the use of a novel wearable device for automatic food intake detection to capture the full range of free-living eating environments of adults with overweight and obesity. In this paper, we document eating environments of individuals that have not been thoroughly described previously in nutrition software as current practices rely on participant self-report and methods with limited eating environment options.

**Methods:**

Data from 25 participants and 116 total days (7 men, 18 women, M_age_ = 44 ± 12 years, BMI 34.3 ± 5.2 kg/mm^2^), who wore the passive capture device for at least 7 consecutive days (≥12h waking hours/d) were analyzed. Data were analyzed at the participant level and stratified amongst meal type into breakfast, lunch, dinner, and snack categories. Out of 116 days, 68.1% included breakfast, 71.5% included lunch, 82.8% included dinner, and 86.2% included at least one snack.

**Results:**

The most prevalent eating environment among all eating occasions was at home and with one or more screens in use (breakfast: 48.1%, lunch: 42.2%, dinner: 50%, and snacks: 55%), eating alone (breakfast: 75.9%, lunch: 89.2%, dinner: 74.3%, snacks: 74.3%), in the dining room (breakfast: 36.7%, lunch: 30.1%, dinner: 45.8%) or living room (snacks: 28.0%), and in multiple locations (breakfast: 44.3%, lunch: 28.8%, dinner: 44.8%, snacks: 41.3%).

**Discussion:**

Results suggest a passive capture device can provide accurate detection of food intake in multiple eating environments. To our knowledge, this is the first study to classify eating occasions in multiple eating environments and may be a useful tool for future behavioral research studies to accurately codify eating environments.

## 1. Introduction

Overweight and obese populations continue to grow throughout the U.S., with currently 73.6% of U.S. adults having overweight or obesity (1). Behavior, environment, and genetic factors all contribute to the rise in overweight and obesity (2). Increasing physical activity and reducing energy intake remain the most common strategies to combat overweight and obesity. However, health extends beyond physical activity and energy intake and novel weight loss strategies are focusing on the broader health spectrum of the individual within their environment (3). The influence of environment on health and behavior can span across social structures, access to food, mental health, socioeconomic status, and a variety of influences among an individual’s daily hierarchy of priority (4, 5).

Eating environments have become of particular interest due to the role of environment on health and health behavior (6–8). Research has shown eating environment can influence energy intake (7), types of foods eaten, and rate of eating (6). Categorizing eating environments has proven challenging thus far for multiple reasons, largely dependent on an individual’s daily routine spanning across multiple environments throughout the day (4). For example, two individuals living in the same neighborhood can have vastly different eating environments based on work time and location, mode of transportation, and extracurricular activities. Social structures (i.e., living alone, with a spouse and/or children) can further complicate how eating environments can be stratified. A growing body of evidence continues to illustrate how eating environments significantly influence amounts and types of food that are eaten (7). Eating while watching the television is an example of an environmental stimulus, which has been shown to increase energy intake, and influences subsequent meals and snacking episodes (7). Watching screens while eating has also been shown to increase the rate of food consumption and reduce the time interval between eating types of food (8). Conversely, increasing the frequency of family mealtimes is positively associated with reduced energy consumption and increased intake of fruits, vegetables, whole grains, and decreased soft drink consumption (9).

Currently, the immediate free-living eating environment is assessed *via* 24-h recall, food record/diary, food frequency questionnaire (FFQ), or a screening questionnaire (10). Common sources of error for these methods are well documented: 24-h recalls provide random error mainly driven by recall bias; food record/diaries provide error from reactivity bias and perceived desirability of answers; FFQs have systematic error driven by lack of detail and estimation of usual intake over a period of time; screener systems have systematic error caused by lack of detail and estimating intakes over periods of time (10). Inconsistencies and inaccuracies often arise due to participant memory and heterogeneity in measures used to assess eating environment (10). Additionally, in contrast to the many validation studies documenting the energy intake accuracy of each method, to our knowledge, there are no data that validate self-report for free living eating environment against an objective measure. Furthermore, biases can emerge when a study participant is required to track meals and eating locations due to their positive and/or negative influences on dietary intake and location (10). Therefore, obtaining a catalog of possible eating environments in free-living situations and codifying eating environments to standardize data analysis has been challenging.

Novel technologies are emerging to passively capture the eating episodes of individuals (11, 12). Camera-based systems (13), body-worn sensors (14), EMG and force sensors (15, 16), and accelerometer-based monitoring systems (17) have all been proposed for passive capture of food consumption, which do not rely on self-report. Novel research involving wearable devices has been developed to alleviate the burden and accuracy problems with self-reported data and offer automatic detection and monitoring of food intake (18). A recent systematic review assessing available methods to automatically detect eating behavior found the use of facial landmarks (i.e., localize and track key points on a human face) is the most promising method for detecting eating events (19). Using novel wearable devices that automatically capture images during food and beverage intake, direct annotation of eating environment can be obtained by capturing meal images, eating location, and who someone is eating with (12). These methods have the advantage of not relying on participant memory or a previously codified but generic and incomplete list of eating environment options (12). Passive intake measurements can improve accuracy of such measurements as they are in free-living conditions and capture data without recall (12).

Our Automated Ingestion Monitor v2 (AIM-2) has the ability to capture images at and around eating episodes under controlled or free-living conditions (20). Our goal is to expand the understanding of eating environments *via* wearable technologies and categorize the full range of free-living eating environments that can build the framework for future behavioral nutrition studies (21, 22).

## 2. Methods

### 2.1. Participants

Data were collected from Brown University and Boston University from November 2020 through August 2021. Data collection dates occurred during several phases of COVID-19 restrictions and reopening phases that varied between both states and by time of data collection. Such restrictions changed over time. For example, limited capacity in restaurants (the lowest being 25% of capacity during some dates at Boston University site) to full capacity, remote work guidance (in office work allowed), and several indoor gathering recommendations (23, 24). The University of Alabama processed eating episode data and the Colorado Clinical and Translational Sciences Institute (CCTSI) Nutrition Core carried out analysis of eating episodes and categorization of eating environments. The Miriam Hospital Institutional Review Board (IRB) approved the study.

Inclusion/exclusion criteria were deliberately broad to recruit a range of participants from different backgrounds. All participants needed to be non-smoking, over 18 years, with a body mass index (BMI) of 27–45 kg/m^2^ and no previously diagnosed medical conditions that could affect their ability to eat or chew food. Participants were openly recruited *via* electronic newsletter on campuses and posted on lab websites. Participants interested in the study completed an online survey link to assess their eligibility and select the Boston or Rhode Island site. Online questionnaires collected basic dietary habits *via* an abbreviated food frequency questionnaire (FFQ) in addition to any food allergies that would influence eating behavior. Self-reported participant demographics, including height and weight to calculate BMI were collected using the online questionnaire. The abbreviated FFQ included details for foods and beverages necessary to assess total energy intake but that are difficult to assess from images, such as type of milk (skim, 2%, whole, or a variant such as soy or almond) usually used, type of salad dressing, and type of butter or mayonnaise used in sandwiches (regular, light, or no fat).

Participants were asked to wear the Automatic Ingestion Monitor (AIM-2) under free-living conditions for at least 12 h per day during waking hours for at least seven consecutive days. No additional instructions were given to participants and they were instructed to go about their normal daily lives. The AIM-2 captures eating episodes in real time within the eye gaze with use of an accelerometer and an optical sensor monitor of the temporalis muscle to detect chewing (20). Placement of the sensor is on the right leg of eyeglasses. Eight-second segments of the sensor signals are processed in real time on the AIM-2 device by a lightweight machine learning classifier based on Linear Discriminant Analysis. For each segment recognized as eating or drinking, the AIM-2 captures 3 consecutive images, each 10s apart. The image capture continues for as long as ingestion is detected, resulting in approximately 3–100 images captured for each eating event. The images and sensor signals are stored internally on an SD card. The data from the SD card are extracted at the end of the study and processed by a more powerful classification algorithm on a stand-alone PC. This algorithm further refines the detection of eating and drinking by eliminating most of the false positives.

An algorithm is used to assess compliance with device wear (25). Participant compliance was defined as a minimum of 8 h of AIM-2 wear time and a minimum of two eating episodes between midnight and 11:59 pm. Only days on which participants were compliant were included for analysis. There were 25 participants (18 female, 7 male), that met criteria with a total of 116 days of analysis. The total days analyzed of compliant wear per participant were: 1 day 20%, 2 days 8%, 3 days 8%, 4 days 8%, 5 days 16%, 6 days 8%, 7 days 12%, 8 days 20%. Three participants did not meet criteria for minimum wear time or minimum number of eating episodes. Participants (18 female, 7 male) had a mean (± SD) age of 44 ± 12 years (range: 18–63 years) and BMI of 34.3 ± 5.2 kg/m^2^ (range: 27.4–44.2). Participants were 60% Caucasian, 16% Hispanic or Latino, 20% Black or African American, and 4% Asian.

### 2.2. Eating environment categories

Eating environments were initially classified using the Nutrition Data System for Research (NDS-R) software version 2021, developed by the Nutrition Coordinating Center (NCC), University of Minnesota, Minneapolis, MN. Upon initial analysis, further expansion from the NDS-R eating environments was warranted, as there were a significant number of eating environments that fell beyond the NDS-R definitions. For example, within the “home” environment, which is a single location category in NDS-R, we observed eating in the living room, bedroom, bathroom, and kitchen. The approach to eating behavior is likely different in each of these home sub-locations, which led to the development of subsets for the “home” category. Five additional sub-locations were added to the home category to include home bedroom, home living room, home (outside), and home dining room. The outside category was defined as any eating episode eaten outside. Eating episodes with a screen were defined as any screen that was used during the eating episode and includes TVs, computers, phones, or tablets. The NDS-R travel category, which combines all modes of transport (car, airport, and train/bus) was expanded as to classify modes individually as car, air, and train/bus (Table 1). This study was conducted during the third wave of COVID, so air travel was rare.

**Table 1:** Number of eating episodes at each meal, stratified to include multiple eating locations during a single eating episode and eating occasions with a screen present.

Location	Breakfast total *n* = 79 *n* (%)	Lunch total *n* = 83 *n* (%)	Dinner total *n* = 96 *n* (%)	Snack total *n* = 218 *n* (%)
With screen	6 (7.6)	12 (14.5)	8 (8.3)	32 (14.7)
Home in dining room	20 (25.3)	16 (19.3)	30 (31.3)	5 (2.3)
Home in living room w/screen	14 (17.7)	5 (6.0)	22 (22.9)	50 (22.9)
Home in dining room w/screen	8 (10.1)	9 (10.8)	13 (13.5)	9 (4.1)
Home in kitchen w/screen	5 (6.3)	4 (4.8)	1 (1.0)	6 (2.8)
Home in kitchen	11 (13.9)	6 (7.2)	4 (4.2)	13 (6.0)
Home in bed w/screen	1 (1.3)	1 (1.2)	1 (1.0)	12 (5.5)
Home in kitchen w/computer	1 (1.3)	0 (0.0)	0 (0.0)	0 (0.0)
Home in living room	0 (0.0)	1 (1.2)	3 (3.1)	8 (3.7)
Home in bed	0 (0.0)	0 (0.0)	1 (1.0)	0 (0.0)
Car	1 (1.3)	9 (10.8)	0 (0.0)	42 (19.3)
Car w/ Screen	2 (2.5)	0 (0.0)	0 (0.0)	2 (0.9)
Restaurant	3 (3.8)	6 (7.2)	5 (5.2)	0 (0.0)
Restaurant w/screen	0 (0.0)	0 (0.0)	3 (3.1)	3 (1.4)
Work in breakroom	0 (0.0)	1 (1.2)	0 (0.0)	10 (4.6)
Work w/screen	3 (3.8)	5 (6.0)	0 (0.0)	12 (5.5)
Outside (home)	0 (0.0)	3 (3.6)	0 (0.0)	0 (0.0)
Outside (not at home)	0 (0.0)	0 (0.0)	2 (2.1)	8 (3.7)
Outside w/ Screen (home)	1 (1.3)	1 (1.2)	0 (0.0)	0 (0.0)
Outside w/screen (not at home)	0 (0.0)	0 (0.0)	0 (0.0)	2 (0.9)
*Multiple locations*				
Home in kitchen and dining room	1 (1.3)	0 (0.0)	1 (1.0)	0 (0.0)
Home in kitchen and living room	1 (1.3)	1 (1.2)	2 (2.1)	1 (0.5)
Home in kitchen and living room w/screen	1 (1.3)	1 (1.2)	0 (0.0)	2 (0.9)
Car and outside (not at home)	0 (0.0)	0 (0.0)	0 (0.0)	1 (0.5)
Restaurant and home w/screen	0 (0.0)	2 (2.4)	0 (0.0)	0 (0.0)

Data shown as number (%). Percent represents the number of eating episodes of that meal type in that location divided by the number of all eating episodes of that meal type (breakfast, lunch, dinner, or snack).

### 2.3. Classification of eating environments by meal type and location

Eating environments were classified manually by examining each image associated with eating episodes. All analysis was conducted within the AIM-2 annotation software (20). Three trained nutritionists from the CCTSI Nutrition Core classified eating environment/s per eating episode. For each day of compliant AIM-2 wear, the date, wear time, number of eating episodes, and eating environment were recorded. If another person was observed in any image/s during an eating episode captured by the AIM-2, they were counted as a person that the meal was eaten with. Any individual that appeared to be under 18 years of age upon visual assessment was counted as a child. As participants are mobile in a free-living setting, multiple eating environments were indicated if a participant changed environment during an eating episode, i.e., started eating in the kitchen but then moved to the living room.

Assessing mealtimes for standard meals (breakfast, lunch, dinner, snack) proved to be difficult as this is highly variable among individuals. *A priori* mealtimes were defined as: Breakfast-6:00 a.m. to 10:00 a.m.; Lunch-12:00 p.m. to 2:00 p.m.; Dinner-6:00 p.m. to 8:00 p.m. Snacks were defined as eating episodes that occurred outside of these times. Upon initial analysis, 26.7% of days had no “meals” as designated by the *a priori* definition of mealtimes but did have multiple snacks throughout the day. A single nutritionist retrospectively analyzed all days with multiple snacks but no designated meals. This involved manual classification into a meal if, for example, the eating episode was outside of the mealtime definition but was a substantial meal, i.e., a large spaghetti entrée, sandwich, pizza, etc. Participant ID, date, eating episode, designation change, and the rationale behind manual change was recorded. A meal was classified as having three or more separate food components, accounting for more than 10% of total number of foods consumed over the day, and the participant had to consume over 25% of the meal. As these variabilities were vital in classifying environments, the dataset with the retrospective analysis were used.

The incidence of eating locations was tabulated descriptively using percent distribution, with the total number of eating episodes for a particular meal type as the denominator. If an eating episode occurred in more than one environment, each environment contributed to the denominator of the calculation for environment specific incidence. For example, if a participant started eating at home in the kitchen then took the meal to their home living room, this would count as two eating environments for a single eating episode.

## 3. Results

**Figure 1:**
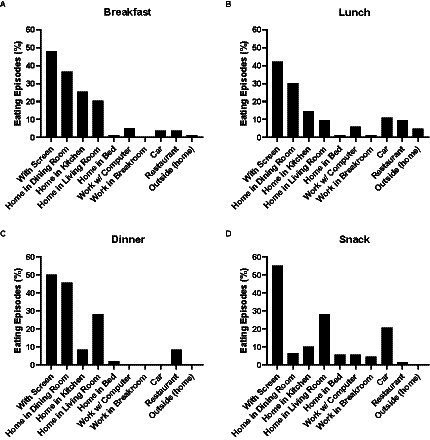
Eating environment distribution (non-stratified) at **(A)** Breakfast; **(B)** Lunch; **(C)** Dinner; **(D)** Snack.

Of the 116  days analyzed, 68% included a breakfast, 72% included a lunch, 83% included a dinner, and 86% included at least one snack. Distribution of breakfast location indicated that 36.7% of eating episodes occurred within the home dining room, 25.3% within the home kitchen, 20.3% within the home living room, with all other categories occurring under 5.1%. In addition, 48.1% of eating episodes occurred with a screen (Figure 1A). Eating episodes in multiple eating environments accounted for 44.3% of all eating episodes. As eating with a screen was tallied as a separate environment, data were further stratified to indicate the distribution of multiple eating environment locations to include eating environments with a screen and/or multiple locations (Table 1). Breakfast eating episodes were mostly eaten alone (75.9%).

Lunch eating episode distribution indicated that 30.1% of eating episodes occurred within the home dining room, 14.5% within the home kitchen, and 10.8% in a car, with all other categories occurring less than 10%. 42.2% of lunches occurred with a screen (Figure 1B). Eating episodes in multiple environments accounted for 28.8% of eating episodes. Data were further stratified to analyze multiple locations (Table 1). Eating lunch alone accounted for 67.5% of all episodes.

Distribution of dinner location indicated 45.8% of eating episodes occurred within the home dining room, 28.1% within the home living room, and all other categories occurring in less than 9% of eating episodes. 50% of dinners occurred with a screen (Figure 1C). Eating environments in multiple places accounted for 44.8% of eating episodes with all data further stratified to indicate multiple environments (Table 1). Eating dinner alone accounted for 74.3% of all dinners.

Snacking episodes occurred 28% within the home living room, 20.6% within a car, with all other categories occurring less than 10.1%. 55% of snacks occurred with a screen (Figure 1D). Eating episodes in multiple environments occurred with 41.3% of all episodes with all data further stratified by locations (Table 1). Eating snacks alone occurred in 74.3% of all snacks.

## 4. Discussion

This proof-of-concept study examined the use of the AIM-2 device to capture the spectrum of eating environments of individuals with obesity in free-living conditions. Results showed that the AIM-2 was successful in capturing substantially more eating environments and providing a richer dataset of combined eating environments compared to NDS-R or any currently available nutrition questionnaire. Results indicated eating at home and with a screen was the most prevalent eating environment among all eating occasions. Of the remaining eating environments, the most common were eating alone, eating at home in the living or dining room, and eating in multiple locations. To our knowledge, this is the first study to classify a single eating episode occurring across multiple eating environments. This may be because this was not previously a codified option in existing nutrition software or questionnaires.

NDS-R is commonly used for eating environment analysis; however, NDS-R meal locations are broad (i.e., home, work, other) and do not capture the nuances of each location (i.e., home – bedroom, bathroom, kitchen, living room, with or without screens, work – breakroom, on laptop, outside, etc.). For example, in the “home” environment, we observed individuals eating in the living room, bathroom, bedroom, and kitchen. This study expanded on codified eating environments and captured significantly more eating environments where an individual consumes food than commonly used software or questionnaires. In addition, other environments not encountered or taken into account in this study are possible, such as school/university, store/mall, etc. Given that this study was conducted during the third wave of COVID, it is possible that future studies may encounter eating environments that we have not categorized. These might include eating at a party or banquet and eating on different forms of public transport. Even though many participants wore the AIM-2 when there were few or no local COVID restrictions, some participants may have chosen to avoid crowded spaces and communal eating. This could be one reason that we observed many meals and snacks being eaten alone. In addition, this study examined only the eating environments of adults with overweight or obesity that cannot be extrapolated to other populations, such as lean adults or children, who eat more often in a family context or in groups at school. Therefore, further research is needed to fully categorize and compare the eating environments of different populations. Data obtained in the current study demonstrate that the AIM-2 device would be valuable for such future research.

The finding of >65% of meals eaten at home in this study differs compared to the National Health and Nutrition Examination Survey (NHANES) data from 2013 to 2014, showing that 50% of daily energy intake occurred outside the home (26). Moreover, the data from the 2011–2018 NHANES showed that 65% US adults consumed ≥2 meals/week outside of the home (27). Our high rate of eating at home may have resulted from public health orders during the coronavirus disease 19 (COVID-19) pandemic. This study was conducted during wave 3 of the pandemic (Nov 2020-Aug 2021) and individuals were confined to their homes at a higher rate than normal for part of this time period. Even after many COVID restrictions were lifted, some participants may have chosen to continue to avoid crowded spaces and communal eating. Future research should build upon this dataset by examining eating environments when no public health restrictions are in place.

We show that 31.9% of days contained no breakfast meal, which is consistent with 2015–2018 NHANES data, showing 15.6% of adults skip breakfast (28). Aurélie Ballon et al. conducted a meta-analysis of 96,175 participants from prospective cohort studies and showed that skipping breakfast 4–5 d/week is associated with a 55% higher risk of developing type 2 diabetes mellitus (T2DM) (29). The high prevalence of snacks throughout the day (85.3% of days with ≥1 snack/day) also is consistent with 2015–2016 NHANES data, showing that 86% of adults reported at least one snack/day, contributing 23% of energy intake throughout the day (30). A review by Skoczek et al. showed the consumption of energy-dense snacks contributes to higher energy intake and body weight in adults (31). The results of this study shed light on snacking occasions and the eating environments they occur in, which commonly include eating in the car. Codifying and defining the eating environment of snacks may provide additional specificity to behavioral interventions aimed at energy restriction for weight loss and/or maintenance.

In this study, more than 50% of all meals were eaten with a screen. Prior to the COVID-19 pandemic, a study had shown 30% of all eating occasions occurring at non-designated eating places, such as with a screen or watching television (32). The high prevalence of eating occasions with screens in this study was not surprising as studies have shown significant increases in screen time and digital device use during the COVID-19 lockdown period across the globe (33, 34). Research from nine European countries involving 4,108 participants found a 65% increase in screen time during the pandemic (33). In a study from Tebar et al., increased screen time during the COVID-19 pandemic was associated with increased sweetened food consumption and increased desire to drink alcoholic beverages (35). Several pre-pandemic studies have shown that energy intake is increased while watching the television (8, 36, 37). Increased screen time during food consumption has been thought to reduce mindful eating. Mindful and intuitive eating constitute an awareness of the present moment while eating (38) and consists of making conscious food choices, an awareness of psychological vs. physical hunger, and eating healthfully in response to those cues (39, 40). The results of this study point to high screen usage, which can reduce mindful eating. This aspect of eating environments, in particular the high prevalence of eating in multiple locations with screens at a single episode, needs to be studied further to determine the direct effects of this behavior on energy and nutrient intake.

This is the first study to examine the prevalence of multiple eating locations during a single meal. Overall, 39.8% of all meals were eaten in multiple locations in and outside of the home. Ogden et al. reported that “eating on the go” disinhibits eating restraint which results in overeating (41). Studies indicate that social interaction may influence eating behavior by increasing meal size and energy intake during meals (42). In this study, 73% of all meals were eaten alone. De Castro et al. found that people consume 50% more food when eating in groups than eating alone (43). The implications of eating alone vs. eating with others in the many locations that we have categorized needs to be studied further.

Strengths of this study include the AIM-2 camera capturing images every 10s when eating was detected, allowing for detection of food intake in multiple eating environments in real-time. The custom-designed software allowed for review of images associated with food intake events as detected by sensors and codifying the eating environments in real time. Limitations of this study include a small sample size and manual codification of eating environments which may lead to errors in location detection. Additionally, the *a priori* designation of mealtimes did not fit one quarter of participants’ circadian patterns. In future studies, this limitation could be addressed by defining meals as a percentage of daily energy or a combination of energy intake and number of food items. Additionally, using the AIM-2 to capture eating episodes has the potential for reactivity bias when participants know that we will be assessing eating habits. It is likely that participant instructions to adhere to their regular activities of daily living, including eating, will not overcome reactivity bias, but wearing the device for multiple days likely decreased the chances that any dietary intake changes would persist throughout the entire period.

## 5. Conclusion

The present study sheds light on the multiple eating environments encountered during free-living that have not previously been documented. Our findings indicate that individuals consumed a majority of meals at home with a screen, alone, and often in multiple rooms during the same eating occasion. Future studies should investigate the correlation of eating environments with energy intake and participant characteristics to facilitate personalized nutrition interventions.

## Data availability statement

The original contributions presented in the study are included in the article/supplementary material, further inquiries can be directed to the corresponding authors.

## Ethics statement

The studies involving human participants were reviewed and approved by University of Rhode Island Institutional Review Board (URI IRB). The patients/participants provided their written informed consent to participate in this study.

## Author contributions

JH, MM, and ES designed the study. MM and GT collected the data. TM, JP, DF, and TG analyzed the data. PZ and WZ performed statistical analysis. JH, MM, GT, TM, MB, JP, and ES provided data interpretation. MB and JP wrote the manuscript draft. All authors reviewed and edited the manuscript and approved the final manuscript. All authors contributed to the article and approved the submitted version.

## Funding

This work was supported by the National Institutes of Health through the National Institute of Diabetes and Digestive and Kidney Diseases award numbers R21DK085462 and R01DK100796 and National Center for Advancing Translational Sciences award number U01TR002535.

## Conflict of interest

GT is a consultant and advisory board member for the Lumme Health, Inc. and the Medifast Inc. MM serves on the Avocado Nutrition Science Advisory for the Haas Avocado Board.

The remaining authors declare that the research was conducted in the absence of any commercial or financial relationships that could be construed as a potential conflict of interest.

## Publisher’s note

All claims expressed in this article are solely those of the authors and do not necessarily represent those of their affiliated organizations, or those of the publisher, the editors and the reviewers. Any product that may be evaluated in this article, or claim that may be made by its manufacturer, is not guaranteed or endorsed by the publisher.
